# The Sincerity of Modern Medicine

**Published:** 1892-06-18

**Authors:** 


### THE DOCTOR'S ARMCHAIR.

### THE SINCERITY OF MODERN MEDICINE.

MOST competent persons have decided, now that Mr. Froude's "Life" has done its best and its worst, that Thomas Carlyle was the greatest writer of his age. He saw deeper, wider, and truer than any of his contemporaries, and he painted his vision more brilliantly and enduringly, and these constitute his right to the first place. There are many who consider the essence of Carlyle's message to be of almost canonical authority. It deserves to be placed side by side with the utterances of sacred books. The soul of the message may almost be concentred in a single word—"Sincerity." The business of Carlyle's life was to be sincere and to make other people so. That was as much his business as it is the business of certain men to build houses, and of certain others to command ships and carry merchandise from port to port. In the judgment of so great a man as Carlyle, it was worth while to devote a whole lifetime, and a powerful intellect to the promotion of the business of "Sincerity."

When we have said of modern medicine that it is eminently and above all things "sincere," we have expressed the conviction that it is one of the worthiest and highest factors of modern civilisation. It is a scientific antiseptic, a preserver, a purifier, a maintainer of the wholesomeness of life in the deepest spiritual, as well as in the material sense. Some may wish for evidence of the truth of our assertion that modern medicine is so pre-eminently sincere. If they are inclined to deny the proposition because certain medical men of their own acquaintance are by no means conspicuously sincere, we would suggest to them that they might as well reason that a whole forest is dead because a few of the outer trees are bare and leafless. In order to get at the actual quality of the medicine of the times we must search out the bedside and laboratory work of its most competent professors, and we must devote study to the literature that grows out of hospital wards and of physiological and pathological laboratories.

The title of this article is suggested by a new monthly magazine—*The Journal of Pathology and Bacteriology*, the first number of which has just been issued. On the back of the magazine are the names of the editor-in-chief, four departmental editors, and sixty-six contributors and collaborateurs; seventy-one names in all. These men are about six times as many as the twelve original Apostles of Christianity, more than six times as many when the name of Judas, the traitor, is left out. It is possible there are Judases among the seventy-one. Some reader may smile at the parallelism here instituted between eleven fishermen and peasants, and seventy-one men of modern culture of more or less scientific eminence. Perhaps, however, one of the best wishes we could offer on behalf of the seventy-one, is that they may do their work as enthusiastically and sincerely as the eleven did theirs; and that the results of their labours may be as enduring, and as great.

A non-medical reader would perhaps be surprised by the titles of some of the articles of this first magazine, and might wonder what they had to do with pathology. The veteran Professor Virchow writes, for example, on "Transformation and Descent," which title has not a very pathological sound to uninitiated ears. The article itself, however, is entirely physiologico-pathological, and profoundly philosophical at the same time. Moreover, and this constitutes an important part of its scientific as distinguished from its clinical value, the article touches the deepest biological problems that are exercising the modern mind at a thousand points. Reading Virchow, one sees that just as a single drop of water, dependent from the tip of a huge icicle, is related not only to the icicle, but to all the snow, the water, and the watery vapour in the world, so also the minutest pathological fact is not only related to the particular structure in which it is observed, but to all disease, and to all organic life.

An article by E. Metchnikoff on "Aqueous Humour, Micro-Organisms, and Immunity," ought to be read by sober and fair minded anti-vaccinators. It would show them that at least one worker in a physiological and pathological laboratory is a man of fine intellect, generous enthusiasm, and transparent sincerity. The mere reading of the article has a purifying and elevating influence on the mind. This at any rate is its effect on one who knows something of the subject. Metchnikoff is of the stuff that single minded martyrs are made of. The Great Paul said many a word that would serve as a motto for a physiological laboratory. One such word was—"This one thing I do: leaving the things that are behind I reach forward to those that are before." A noble utterance of a noble intellect! This is the kind of motto that seems to inspire Metchnikoff, and men of like mind.

In a short article like the present it is impossible to give anything like even an outline of the eleven contributions by competent men which constitute the first number of the *Journal of Pathology and Bacteriology*. Our object in drawing attention to the new magazine is to impress upon general readers the fact that modern medicine is a deeply earnest and competent worker at its own particular portion of the almost overwhelming problem which is set before us in organic life and destiny. That may seem a somewhat vague purpose, but we have another which is more immediately definite. It is fundamentally important, both to medical men and to civilisation, that the magazine should live and become growingly vigorous. It cannot live without support. The most cultured medical men will buy it as a matter of course; but all doctors ought to buy it, even those who can hardly spare time to read it. They ought to buy it, because of the illumination and help which it will give directly to its studious readers, and indirectly to medicine as a whole. Every hospital, great and small, ought also to buy it; in the first place for the use of its resident medical officers, and in the second in order to contribute something to its continued life and strength. The magazine is in no sense a mere professional journal. It is the periodical record of a part of the living scientific conflict which the earnest modern man is waging with the world in which he lives. For this sincere scientifico-medical journal, no more fitting editor could have been found than Dr. German Sims Woodhead to guide its fortunes.

